# *Staphylococcus aureus* Panton-Valentine Leukocidin Contributes to Inflammation and Muscle Tissue Injury

**DOI:** 10.1371/journal.pone.0006387

**Published:** 2009-07-27

**Authors:** Ching Wen Tseng, Pierre Kyme, Jennifer Low, Miguel A. Rocha, Randa Alsabeh, Loren G. Miller, Michael Otto, Moshe Arditi, Binh An Diep, Victor Nizet, Terence M. Doherty, David O. Beenhouwer, George Y. Liu

**Affiliations:** 1 Division of Pediatric Infectious Diseases and the Immunobiology Research Institute, Cedars-Sinai Medical Center, Los Angeles, California, United States of America; 2 Department of Pediatrics, David Geffen School of Medicine, University of California, Los Angeles, California, United States of America; 3 Division of Infectious Diseases, Veterans Affairs Greater Los Angeles Healthcare System, Los Angeles, California, United States of America; 4 Department of Medicine, David Geffen School of Medicine, University of California, Los Angeles, California, United States of America; 5 Department of Pathology and Laboratory Medicine, Cedars-Sinai Medical Center, Los Angeles, California, United States of America; 6 Division of Infectious Diseases, Harbor-University of California-Los Angeles, Torrance, California, United States of America; 7 Laboratory of Human Bacterial Pathogenesis, National Institute of Allergy and Infectious Diseases, The National Institutes of Health, Bethesda, Maryland, United States of America; 8 Division of Infectious Diseases, Department of Medicine, University of California, San Francisco, California, United States of America; 9 Department of Pediatrics and Skaggs School of Pharmacy & Pharmaceutical Sciences, University of California, La Jolla, California, United States of America; Charité-Universitätsmedizin Berlin, Germany

## Abstract

Community-associated methicillin-resistant *Staphylococcus aureus* (CA-MRSA) threatens public health worldwide, and epidemiologic data suggest that the Panton-Valentine Leukocidin (PVL) expressed by most CA-MRSA strains could contribute to severe human infections, particularly in young and immunocompetent hosts. PVL is proposed to induce cytolysis or apoptosis of phagocytes. However, recent comparisons of isogenic CA-MRSA strains with or without PVL have revealed no differences in human PMN cytolytic activity. Furthermore, many of the mouse studies performed to date have failed to demonstrate a virulence role for PVL, thereby provoking the question: does PVL have a mechanistic role in human infection? In this report, we evaluated the contribution of PVL to severe skin and soft tissue infection. We generated PVL mutants in CA-MRSA strains isolated from patients with necrotizing fasciitis and used these tools to evaluate the pathogenic role of PVL *in vivo*. In a model of necrotizing soft tissue infection, we found PVL caused significant damage of muscle but not the skin. Muscle injury was linked to induction of pro-inflammatory chemokines KC, MIP-2, and RANTES, and recruitment of neutrophils. Tissue damage was most prominent in young mice and in those strains of mice that more effectively cleared *S. aureus*, and was not significant in older mice and mouse strains that had a more limited immune response to the pathogen. PVL mediated injury could be blocked by pretreatment with anti-PVL antibodies. Our data provide new insights into CA-MRSA pathogenesis, epidemiology and therapeutics. PVL could contribute to the increased incidence of myositis in CA-MRSA infection, and the toxin could mediate tissue injury by mechanisms other than direct killing of phagocytes.

## Introduction

Although previously confined to hospitals and nursing homes, methicillin-resistant *Staphylococcus aureus* (MRSA) has encroached upon immunocompetent populations and poses a growing threat to public health worldwide [1]–[4]. The Panton-Valentine leukocidin (PVL) is a two-component (LukS-PV and LukF-PV) pore-forming toxin secreted by most CA-MRSA strains with demonstrated *in vitro* activity against human leukocytes in its purified form [5]. The toxin is linked in multiple clinico-epidemiological studies to unusually severe disease pathology [6]–[9], especially in young, previously healthy hosts [8], [9], an association that has earned PVL the unproven and controversial reputation of being the major virulence determinant of severe CA-MRSA infections such as necrotizing pneumonia, myositis and necrotizing fasciitis. The virulence of PVL has been formally studied in the laboratory using isogenic *S. aureus* strains (with or without PVL) in murine models of skin infection and necrotizing pneumonia [10]–[15]. However, the published results from multiple groups have been strongly conflicting. In one notable study, introduction of the *lukSF-PV* genes into a PVL^−^ *S. aureus* laboratory strain significantly enhanced pathogenic potential in a mouse pneumonia model [12]. By contrast, deletion of *lukSF-PVL* from the genome of two CA-MRSA strains, MW2 (USA400) and LAC (USA300) had no impact on virulence of the strains in murine models of skin, lung, and bloodstream infection in several published studies [10]–[13], [15]. These subsequent investigations dampened enthusiasm for PVL as a major virulence determinant of CA-MRSA infections.

One caveat regarding analysis of PVL in small animal models is a demonstrable species specificity of toxin susceptibility; for example, human cells have been reported to be 10-fold more susceptible to PVL-mediated lysis than mouse cells *in vitro* [34]. Two groups have reported that administration of microgram amounts of purified PVL toxins under the skin of rabbits or into the lung of mice produced significant pathology and inflammation [12], [16]. The findings suggest that PVL expression by live CA-MRSA strains during mouse or rabbit infection could be below a critical threshold for tissue injury, thus accounting for the lack of a consistent PVL-related pathology. To probe this possibility, we investigated in a mouse skin and soft tissue infection model whether administration of a higher inoculum of CA-MRSA would uncover a PVL virulence phenotype.

## Results

### PVL contributes to muscle but not skin injury

**Figure 1:**
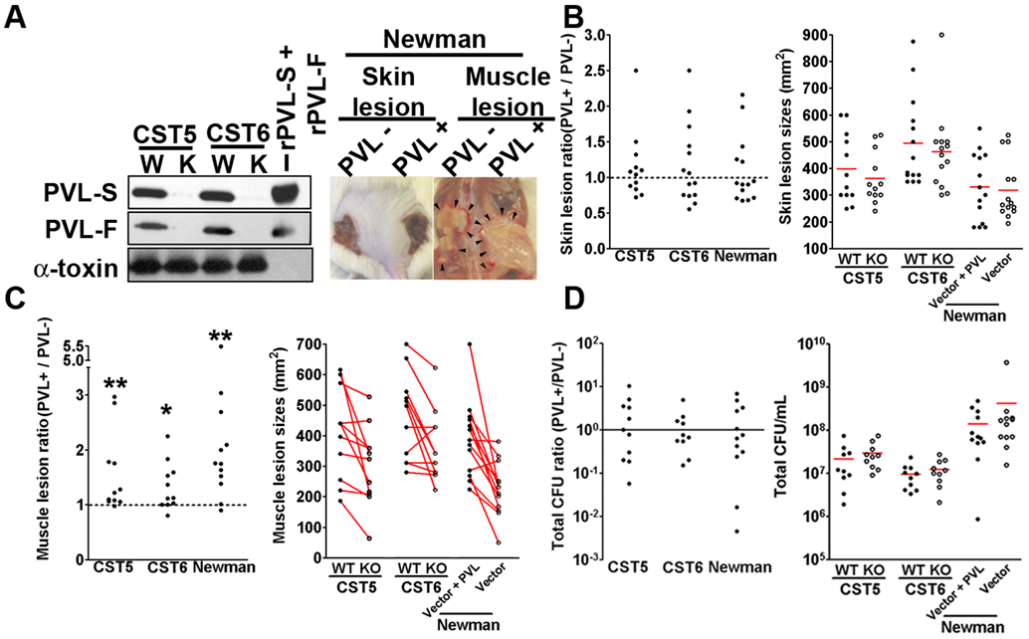
PVL promotes muscle injury. CD1 mice were injected subcutaneously with 10^9^ PVL^+^ *S. aureus* (Newman+PVL vector, CST5, CST6) on one flank, and 10^9^ isogenic PVL^−^ *S. aureus* (Newman+empty vector, PVL^−^ CST5, PVL^−^ CST6) on the opposite flank. Mice were sacrificed 3 days post-infection. For data set B–D, graphs on the left display ratios of lesion sizes or ratios of CFU (PVL^+^∶PVL^−^) based on measurements from each individual mouse; graphs on the right show lesion sizes or CFU grouped according to bacterial strains. (A) Left: Western blot showing expression of LukF-PV, LukS-PV, and α-toxin by CST5+/−PVL and CST6+/−PVL; right: Representative images of skin and muscle lesions. Arrows point to muscle lesion. (B) Skin lesion sizes (C) muscle lesion sizes. (D) Total tissue CFU. In 1C (right panel), muscle lesions harvested from the same mice are joined by a line. * *p*<0.05, ** *p*<0.01.

Bacterial strains applied to our infection model included two PVL^+^ USA300 strains isolated from wounds of patients with necrotizing fasciitis (CST5 and CST6), a PVL^−^ strain (Newman) and PVL^+^ or PVL^−^ isogenic strains engineered from these bacteria [22] (Table S1). A Western blot confirming PVL expression by PVL^+^ strains, but not PVL^−^ strains, is shown in Figure 1A.

Previous investigations have demonstrated that a minimum inoculum of 10^7^ bacteria is typically required to induce a consistent skin lesion in mice [10], and inocula between 10^7^ and 10^8^ CFU have been routinely used in *S. aureus* skin infection studies. To determine whether a higher inoculum would unveil a PVL virulence role, CD1 mice were inoculated subcutaneously on one flank with 10^9^ CFU of a PVL^+^ strain, and on the opposite flank with an equal inoculum of an isogenic PVL^−^ strain. The mice were euthanized on day 3 for analysis of lesions. As shown in Figure 1A and B, mice exhibited no differences in skin lesion size at the site infected with PVL^+^ or PVL^−^ strains. Unexpectedly however, PVL^+^ strains induced larger muscle lesions compared to isogenic PVL^−^ strains (Figure 1A and C), and complementation of the mutant with a PVL expression vector restored the ability to cause more severe muscle injury (Figure S2A). The number of bacteria recovered from lesions produced by PVL^+^ and PVL^−^ bacteria were comparable (Figure 1D and S2B); hence the increased lesion severity associated with PVL^+^ strains could not be explained by increased bacterial survival *in vivo*. CD1 mice, infected with 10^7^ or 10^8^ PVL^+^ or PVL^−^ isogenic strains, showed no difference in muscle tissue injury (Figure S1).

### Histology and PVL expression in infected tissue

To determine whether PVL^+^ and PVL^−^ strains contribute to differences in tissue histology, H&E stain was performed. Overall, H&E stain showed marked necrosis and neutrophil infiltration within the central focus of infection produced by PVL^+^ or PVL^−^ strains, but beyond lesion size differences, a clear-cut difference in pathology was not discernable (Figure S3).

**Figure 2:**
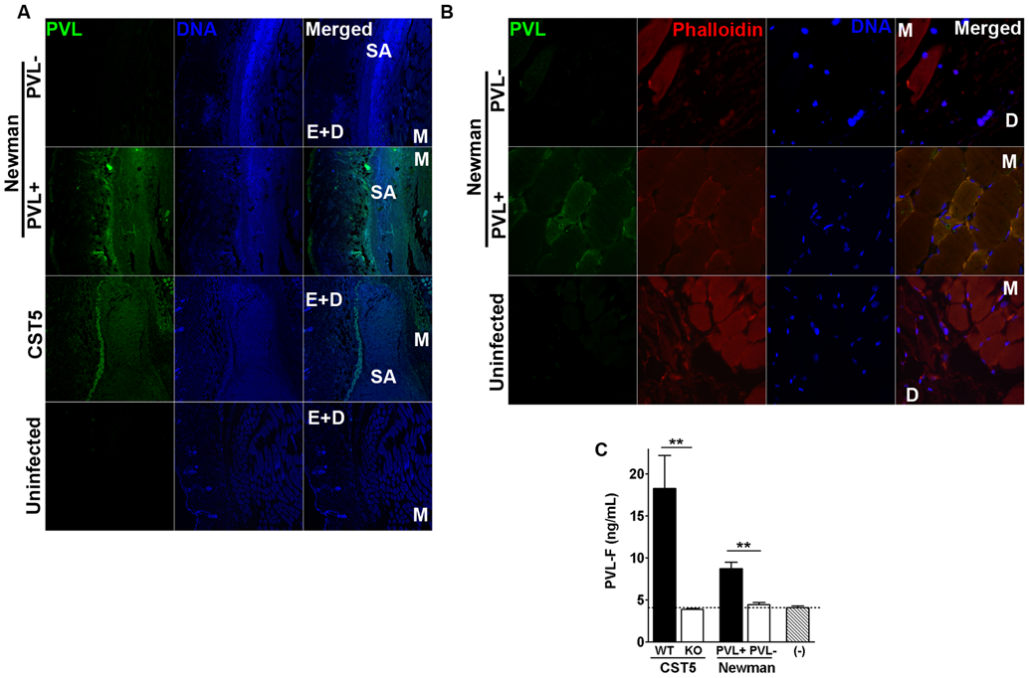
PVL expression in infected tissues. CD1 mice were injected subcutaneously with 10^9^ PVL^+^ *S. aureus* (Newman+PVL vector, CST5) on one flank, and 10^9^ isogenic PVL^−^ *S. aureus* (Newman+empty vector, PVL^−^ CST5) on the opposite flank. (A) PVL immunofluorescence staining of uninfected and infection tissues at 72 h post-infection. Left panels: tissues were stained with PVL-FITC; middle panels: DNA was stained with DAPI; right panels: PVL and DAPI stains were merged. E+D:epidermis-dermis layer, SA: *S. aureus*, and M:muscle. (B) PVL immunofluorescence staining of uninfected and infection tissues. Shown are PVL-FITC, phalloidin conjugated to Texas Red, and DAPI (DNA) stainings of tissues collected at 30 minutes post-infection. D: dermis, and M:muscle. (C) Level of tissue PVL measured by ELISA at 72 h post-infection. ** *p*<0.01.

To evaluate PVL expression in injured tissues, infected skin and muscle were excised for PVL immunofluorescence and histologic analyses. As evidenced by diffuse staining throughout the tissue slices, the PVL^+^ necrotizing fasciitis clinical isolate (CST5) and PVL^+^ Newman strain both expressed the toxin *in vivo* (Figure 2A). PVL staining was particularly prominent around PVL^+^ *S. aureus* clusters, but was also noted on select muscle bundles, particularly at early time points of infection (Figure 2A and B). Based on measurement of PVL concentration by ELISA (Figure 2C), PVL^+^ Newman secretes lower levels of PVL compared to CST5, even though Newman showed a more prominent PVL effect on muscle injury compared to CST5. The measured toxin level in tissues was in the range of 10–20 ng/ml at day 3, and never significantly exceeded those levels at earlier time points based on a time course experiment performed using PVL^+^ Newman (data not shown). Of note, the ELISA is likely to underestimate the actual PVL concentration in infected tissue since much of the toxin that intercalated into host cell membranes might not be have been adequately solubilized to permit measurement in an ELISA.

### PVL-specific antibodies block PVL mediated muscle injury

**Figure 3:**
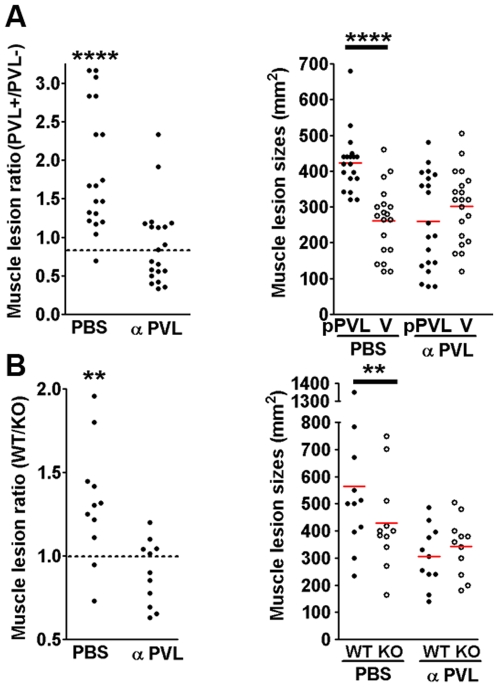
Anti-PVL antibodies ameliorate severity of muscle injury. CD1 mice were injected intraperitoneally with DPBS or rabbit antisera against LukS-PV and LukF-PV. Twenty four hours post-injection, mice were infected on opposite flanks with either PVL^+^ or PVL^−^ isogenic *S. aureus*. Shown are ratios of lesion sizes (PVL^+^∶PVL^−^) and muscle lesion sizes from individual mice on day 3 post-infection. Bacterial inocula were (A) Newman+/−PVL, and (B) CST6+/−PVL. ** *p*<0.01.

To investigate whether blockade of PVL could reduce the extent of muscle injury, mice were injected intraperitoneally with PVL-specific antibodies or DPBS, then challenged the next day with ∼10^9^ bacteria. As shown in Figure 3A and B, pretreatment with PVL-specific antibodies significantly reduced the size of muscle lesions induced by PVL^+^ strains, but did not alter lesion sizes caused by PVL^−^ strains. Results of these experiments corroborate the pathogenic role of PVL in severe soft tissue infections in the mouse model, and once again indicate that damage inflicted by PVL predominantly affects deep muscle tissues but not superficial skin layers.

### PVL contributes to inflammation

**Figure 4:**
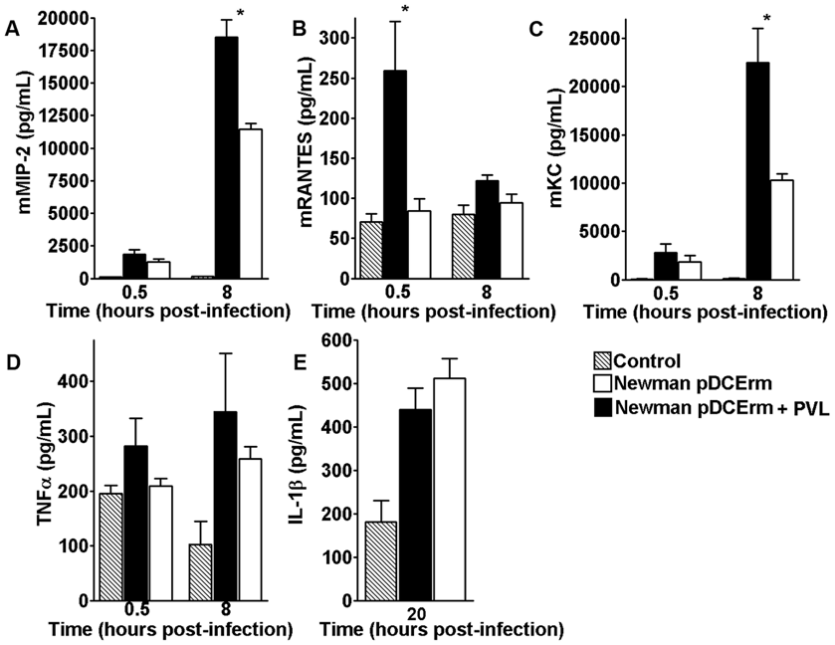
Tissue chemokines and cytokines after infection with *S. aureus* Newman (+/−PVL). CD1 mice were infected on opposite flanks with either Newman+empty vector or Newman+PVL expression vector. Infected tissues were harvested at the indicated time post-infection and tissue chemokines and cytokines were measured by ELISA. (A) Tissue MIP-2, (B) RANTES,(C) KC, (E) TNF-α, and (D) IL-1-β. Controls consisted of DPBS injected mice. * *p*<0.05.

It is well established that host inflammation can have devastating consequences during infection [23]. Because PVL cytolysis of PMN is difficult to demonstrate *in vitro* or *in vivo* using live bacteria, we next asked whether PVL induces inflammation during infection, by excising lesions from infected CD1 mice and measuring expression of select cytokines and chemokines by ELISA. As shown in Figure 4A–C, PVL-expressing *S. aureus* Newman induced higher levels of chemokines KC, MIP-2, and RANTES in the injured tissues than its isogenic PVL- mutant; no differences in TNF-α and IL1β induction were observed between the two strains (Figure 4D and E). These results are consistent with report by Konig and coworkers that sublytic PVL toxin concentration could induce secretion of IL-8 (human equivalent of KC) by PMN [24].

### Effect of host genetic background on PVL virulence

**Figure 5:**
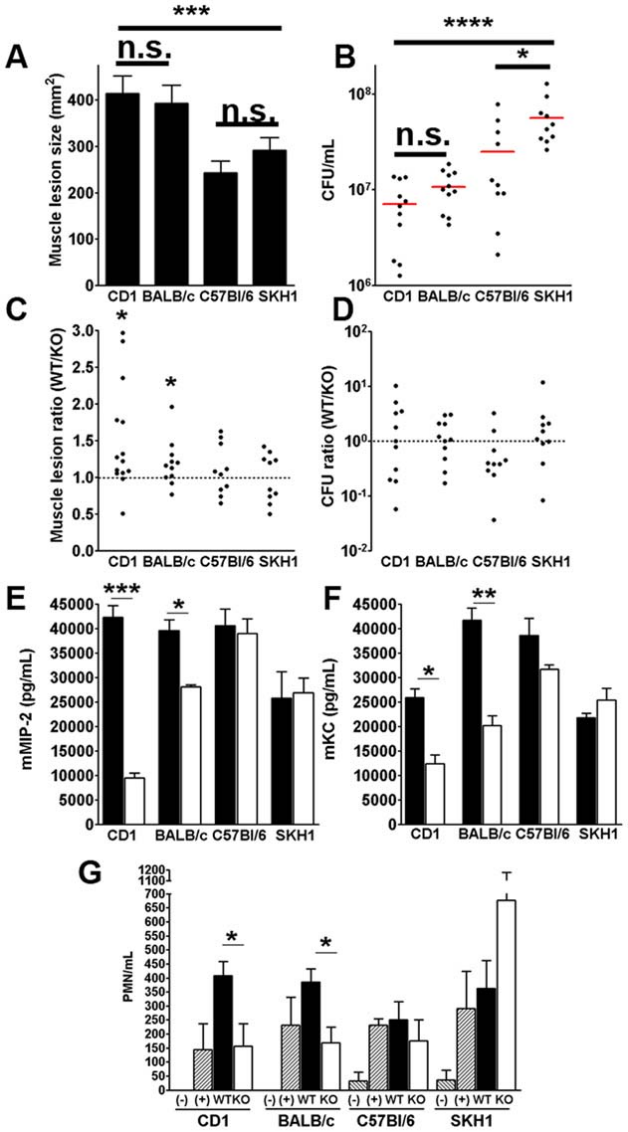
Effect of innate immunity and host background on PVL virulence function. Ten to twelve week old CD1, C57BL/6, BALB/c, and SKH1 mice were infected on opposite flanks with either PVL^+^ CST5 or PVL^−^CST5 *S. aureus*. Mice were sacrificed at different time points for analyses. (A–D) Effect of host background on muscle pathology and total tissue CFU 3 days post-infection with CST5+/−PVL. (A) and (B) Plot of muscle lesion sizes and total tissue CFU after infection with WT CST5. (C) and (D) plots of lesion size ratios and CFU ratios (PVL^+^∶PVL^−^) from individual mice. (E) and (F) Effect of host background on tissue chemokine levels 8 h post-infection with CST5+/−PVL. (G) Effect of host background on tissue MPO activity 3 h post-infection with CST5+/−PVL. Controls consisted of DPBS injected mice (negative control) and LPS injected mice (positive control). * *p*<0.05, ** *p*<0.01, *** *p*<0.005, **** *p*<0.001.

Next, to facilitate studies of immune response to PVL, we repeated the infection experiments in C57/B6 mice, the background mouse strain on which most knockouts are maintained. Injection of C57/B6 mice with 10^9^ WT CST5 and PVL^−^CST5 mutant, unexpectedly, elicited muscle lesions of comparable size (Figure 5C), suggesting that the mouse's genetic background is a further determinant of PVL-induced disease pathology. Previously, Bubeck Wardenburg and colleagues have demonstrate that host background differences could be a determinant of PVL virulence [11], [13]. In their study, PVL showed no virulence effect in C57BL/6 mice [11] but paradoxically attenuated pathogenicity of *S. aureus* in BALB/c AnNHsd mice [13]. To further evaluate whether and how the mouse genetic background and immune system contribute to PVL mediated injury, we examined skin and soft tissue infection in four different mouse strains: BALB/c, C57BL/6, SKH1, and CD1. As shown in Figure 5C, PVL contributed to muscle lesions in CD1 and BALB/c mice, but had no effect on lesion severity in SKH1 and C57BL/6 mice. The presence of PVL related injury in any particular strain of mouse correlated directly to differences in PVL-associated chemokine secretion (Figure 5E and F). Specifically, the CST5 PVL^+^ strain elicited significantly higher levels of MIP-2 and KC in CD1 and BALB/c mice compared with the isogenic PVL^−^ strain, but this chemokine differential did not occur in SKH1 or C57BL/6 mice. Furthermore, as measured by tissue MPO activity, PVL induced increased neutrophil infiltration in CD1 and (to a lesser extent) in BALB/c mice at 3 h post-infection, but not in SKH1 and C57BL/6 mice (Figure 5G). No difference was observed between WT *S. aureus* and the isogenic PVL knockout mutant when MPO was measured at 12 h (Figure S4B). Overall, these results suggest that PVL induction of host immune responses depends on the genetic background of the mouse. We noted interestingly that CD1 and BALB/c mice (+PVL phenotype) cleared WT CST5 infection much more effectively compared to SKH1 or C57BL/6 mice (no PVL phenotype), but developed significantly larger lesions compared to SKH1 or C57BL/6 mice (Figure 5A and B). A possible interpretation of these findings could be that CD1 and BALB/c mice detect and respond to PVL with an exaggerated pro-inflammatory reaction and neutrophil recruitment, which achieves more rapid bacterial clearance, but at a simultaneous cost of more extensive collateral damage to host tissues.

### Pathogenic effects of PVL in older mice

**Figure 6:**
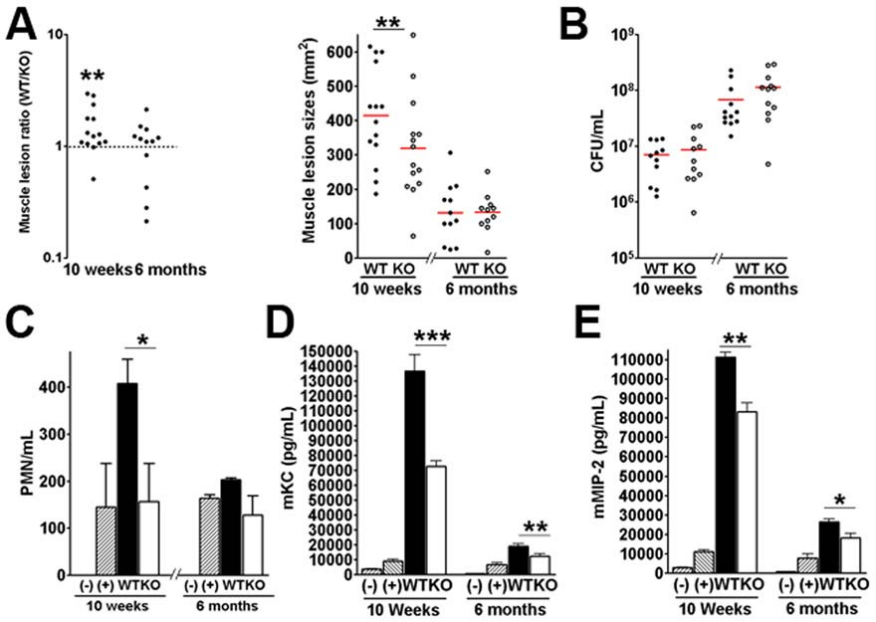
Effect of innate immunity and mouse age on PVL virulence function. Ten to twelve week old CD1 mice were infected on opposite flanks with either PVL^+^ CST5 or isogenic PVL^−^ CST5. (A) Muscle lesions, (B) total tissue CFU 3 days post-infection, (C) tissue MPO activity at 3 h post-infection, and (D) and (E) KC and MIP-2 levels at 3 h post-infection. * *p*<0.05, ** *p*<0.01, *** *p*<0.005.

Previous epidemiologic studies have demonstrated an association between PVL and severe infections among young and immunocompetent individuals [8], [9]. To examine whether pathogenic effects of PVL vary with age, 6 month-old mice were injected subcutaneously with isogenic PVL^+^/^−^ CST5 strains. As shown in Figure 6A–E, the 6 month old mice had significantly smaller muscle lesions, reduced chemokine secretion, but had a much higher tissue bacterial load compared to 10–12 week old mice. In older mice, PVL had no impact on tissue injury, neutrophil recruitment, or surviving CFU (Figure 6A, B and C), but had a small but significant impact on chemokine secretion (Figure 6D and E). These results are consistent with findings that older individuals have more limited immune response to bacterial challenge [25]–[28], and have less severe pathology associated with PVL [8], [9].

## Discussion

Multiple epidemiologic studies have provided compelling evidence linking PVL to pathogenicity of *S. aureus* infections [6], [7], [29]–[31]. Notably, an *S. aureus* strain responsible for an epidemic in the 1950's (phage type 81/80) also harbored the LukFS-PV genes; the strain mysteriously disappeared following the introduction of methicillin [32], but PVL resurfaced in the 1990's in association with severe cases of necrotizing pneumonia and furunculosis in MSSA and CA-MRSA strains [8], [33]. However whether PVL itself is a causative factor responsible for increased disease severity has been heatedly debated because of failure by multiple groups to demonstrate that PVL has a virulence role [10]–[15]. More specifically, in the skin infection model, Voyich et al. and subsequently Bubeck Wardenburg et al. infected C57BL/6 and BALB/c mice with 10^7^ WT or PVL KO in the *S. aureus* LAC USA300 background. In both studies, the authors report either no difference in skin lesion size (in BALB/c or C57BL/6 mice) or a larger skin lesion (in BALB/c AnNHsd mice) inoculated with the PVL mutant strain. The authors did not evaluate muscle lesions in these studies. Brown and coworkers performed an infection experiment using the same inoculum and bacterial strains, and reported a visual difference in muscle lesion size on day 7, albeit the lesion sizes were not quantitated [14]. Here, in a model of severe soft tissue infection achieved using a higher *S. aureus* inoculum, we show that PVL contributes significantly to severity of muscle tissue pathology.

Whether PVL would have a more dramatic effect in human infection will undoubtedly be a focal point of continued debate. At issue are 1) the sensitivity of the mouse tissues to PVL compared to human tissues and, 2) the level of PVL expressed in mouse versus human tissues during CA-MRSA infections. Szmigielski and coworkers have reported that mouse phagocytes are less susceptible to lysis compared to human phagocytes and show an approximate ten fold difference in membrane permeability following purified PVL toxin challenge as measured by 51-Cr release assay [34]. Additionally, we have found in our study that the concentration of PVL measured in mouse tissue, following subcutaneously infection with 10^9^ PVL^+^ CA-MRSA, is in the range of 10–20 ng/ml, which even with an underestimate of the PVL concentration in infected tissue for technical reasons (see Results), is still 50–100-fold lower than PVL measured from human abscess samples (median∼>1 µg/ml) [35]. The possibility of differential PVL induction in human and mouse tissues in human and mouse response is another potential confounding factor in extrapolating animal data to the human condition. Ultimately, the role of PVL in human diseases will need to be addressed by the use of humanized animals, or possibly by the therapeutic effect of PVL-specific antibodies on human CA-MRSA infections.

Notwithstanding the differences between human and mouse infection, our model has features that mimic the human disease. PVL in the mouse preferentially causes injury of the underlying muscle following subcutaneous CA-MRSA injection, and histologic evaluation showed moderate staining of muscle tissues with anti-PVL antibodies. Consistent with this finding, we have recently submitted a report of a child with myositis caused by a PVL^+^ methicillin-sensitive *S. aureus* strain, whose muscle tissue stained selectively and strongly with a PVL specific antibody. It is not known how PVL binding to muscle tissues or PVL induced chemokines contribute to muscle injury. However, given the reported association of severe myositis with CA-MRSA and PVL, the parallel findings in human and mouse strongly suggest a causal link between PVL and human myositis [36].

Multiple studies point to a strong association between PVL and severe necrotizing pneumonia and furunculosis in the current CA-MRSA epidemic [8], [9]. These infections appear to target young and healthy hosts, who appear to suffer more severe infection compared to older or immunosuppressed individuals. Consistent with these findings, we observed that PVL induced greater inflammation and caused greater pathology in mice without conferring a bacterial survival advantage. Further, a PVL effect on tissue pathology was apparent in those mice with the most effective immune clearance of *S. aureus* (10–12 week old CD1 and BALB/c mice), but was insignificant in mice that had a more limited response to the pathogen (C57BL/6, SKH1, and 6 month old CD1 mice). These findings, when taken together with the cytokine results, suggest a parallel between our model and the human infection, and suggest two conclusions: 1) in CA-MRSA, a primary pathogenic effect of PVL is to provoke an overly-robust inflammation, and 2) the severity of PVL-specific pathology may ultimately depend on the capacity of the host immune system for mounting an aggressive neutrophil response to infection. These results are consistent with human epidemiologic reports indicating that younger, immunocompetent individuals infected with MRSA are more susceptible to severe injury [8], [9], and suggest that individuals with enhanced immune response to pathogens are at higher risk for more serious pathology.

Prior efforts to link PVL to human pathology have focused primarily on the cytolytic properties of PVL. Lysis of human phagocytes could be readily demonstrated using purified PVL [34], but a study conducted using live WT and PVL knockout *S. aureus* showed no lytic effect of the toxin on human neutrophils across a range of bacterial concentrations [10]. Our study suggests that a function of PVL more readily achievable at physiologic doses is its ability to provoke inflammation and recruit immune effector cells such as neutrophils through activation of inflammatory molecules (e.g., RANTES, KC, and MIP-2). *In vitro*, PVL induction of IL8 could be demonstrated using human neutrophils at a concentration lower than that required to induce cell lysis [37]. These findings suggest that PVL induced PMN cytolysis as the primary explanation of PVL injury may need to be reevaluated. Under our model, enhanced inflammation, particularly recruitment of phagocytes, could explain PVL-associated “spider bite” lesions and abscesses (representing phagocyte accumulation), which have become the most common presentation of CA-MRSA skin and soft tissue infections [38]. Though our study highlighted differences in chemokine induction by PVL^+^ and PVL^−^ strains, PVL likely induces additional pro-inflammatory factors during infection. In tissue culture, Konig and coworkers have shown that PVL triggers secretion of leukotriene B4 and oxygen metabolites when incubated in the presence of human neutrophils [37]. Hence, inflammation initiated by multiple pro-inflammatory mediators is likely an important contributor to PVL-mediated pathology in our model.

Finally, our data suggest that application of antibodies against PVL could limit or even abrogate PVL-mediated injury. However, anti-PVL antibodies may not be effective for all CA-MRSA infections. In preliminary experiments, we have tested few additional CA-MRSA WT and PVL KO strains (LAC and MW2), but found that under our experimental conditions, PVL expressed by LAC and MW2 was associated with increased bacterial survival but similar level of muscle tissue injury on day 3 (unpublished data). We are performing more exhaustive studies in mice to identify conditions under which PVL expressed in other *S. aureus* backgrounds would induce muscle injury.

In summary, we have developed a model of severe necrotizing soft tissue infection in which PVL shows significant contribution to muscle injury. Though the model does not by itself resolve the debate on the relative importance of PVL in the MRSA epidemic, it unveils surprising parallels between the mouse and human disease, and provides novel insights towards PVL related immunopathology; hence the model could prove to be valuable for further investigation of PVL functions.

## Materials and Methods

### Bacterial strains and growth conditions

Two clinical CA-MRSA isolates (CST5 and CST6), their isogenic PVL knockout strains, one laboratory strain (Newman) transformed with either an empty vector or a PVL expression vector were used for this study (Table S1). The PVL knockout of CST5 and CST6 isolates were constructed by site-directed mutagenesis using primer pairs GAAAGGAAATGATTTTTAGGTC, GACCTAAAAATCATTTCCTTC, AATATTCTATTGGAAAGGCCACC and CTCAATATTGTTATCAGCTTTAG to introduce two stop codons in the *lukS-PV* open reading frame (ORF). The DNA fragments containing the two stop codons were cloned into pMAD [17], and then electroporated into RN4220. The mutant allele was transduced into CST5 and CST6 using phage 80α using a previously established protocol [18]. For complementation and overexpression studies, the PVL locus was amplified by PCR using primer pair CTGACCGCGGTATGACGGCGCATATT-GTATCAATG, and GCCAGGATCCCACGTCAATTAAGACGTGGTTACCC and cloned into shuttle vectors (Table S1). *S. aureus* strains were routinely cultured on sheep blood agar plates and colonies with comparable hemolysis phenotypes were selected for each experiment. Bacteria were also cultured in Todd-Hewitt broth or L- broth at 37°C with shaking at 250 rpm.

### Cloning and expression of rLukF-PV and rLukS-PV

*lukS-PV* and *lukF-PV* were amplified by PCR using flanking primer sequences (*lukS-PV*: CACCGAATCTAAAGCTGATAACAAT and TCAATTATGTCCTTTCACTTTAATTTC; *lukF-PV* CACCGCTCAACATATCACACCTGT and TTAGCTCATAGGATTTTTTTCCTTAG) and cloned into pET151/TOPO-D (Invitrogen). Recombinant PVL proteins were expressed in *E. coli* at 30°C in the presence of 1 mM IPTG, His_6_-tagged proteins were purified over nickel/cadmium columns, and quantitated by BCA (Pierce). The His_6_-tag was removed by AcTEV protease per manufacturer's protocol (Invitrogen), with cleavage confirmed by SDS-PAGE.

### Generation of rabbit antisera

Rabbits were hyperimmunized with 100 µg recombinant LukS-PV or LukF-PV protein in emulsion with Freund's adjuvant to generate specific, high-titer antisera to both LukS-PV and LukF-PV. For each protein, two rabbits were immunized. After 4 immunizations (on days 0, 7, 21, and 35) specific antibody titers increased 500,000-fold for both rabbits immunized with rLukF-PV and >2,000,000-fold for one rabbit immunized with rLukS-PV as measured by ELISA in which plates were coated with LukS-PV 10 µg/mL. The sera were tested against alpha-hemolysin or gamma-hemolysin in Western blot and ELISA and did not show cross-reactivity to either.

### Murine skin infection model

Ten week old SKH1, CD1, and BALB/c mice and 6 month old CD1 mice were purchased from Charles River Laboratories. C57BL/6 mice were obtained from The Jackson Laboratory. Overnight bacterial culture was diluted 1∶1000 in prewarmed media and incubated at 37°C with shaking at 250 rpm until an A_540_∼2.5. Bacteria were harvested by centrifugation at 4000 rpm for 10 min at 4°C, and then washed twice with equal volume of DPBS (Mediatech). The bacteria were resuspended in DPBS at a concentration of ∼10^8^–10^10^ CFU/mL (±20%), and 100 µL of suspension were injected subcutaneously in each flank. Injections were performed with careful visualization of the needle to assure that the injection is not intramuscular.

For experiments assessing the therapeutic efficacy of anti-PVL antibodies, 1 mL of rabbit antisera against LukS-PV and LukF-PV, or DPBS was administered intraperitoneally at 24 h prior to infection. All animal experiments were approved by the Cedars-Sinai Committee on the Use and Care of Animals and performed using accepted veterinary standards.

### Determination of lesion size and tissue bacterial CFU

Following euthanization, skin and muscle lesions were measured and both tissues were excised separately and homogenized in 1 mL of DPBS for CFU determination. The homogenized suspension was centrifuged at 15,000×*g* for 10 min and supernatants were stored at −80°C for subsequent analysis by ELISA. For the skin, lesions were defined by darkened areas of necrosis; for muscle, the lesions consisted of raised pale or darkened colored lesions overlying the red colored healthy tissue. An area of hyperemia is often visualized around the muscle lesions. Muscle lesions are further differentiated from occasional fat tissue based on color and consistency.

Our method to measure lesion size has been previously reported [19]. Both skin and muscle lesions were quantitated by multiplying the length and width of the lesion. Irregularly-shaped lesions were broken down into smaller symmetrical pieces, and each piece was measured by the same method. Thickness and weight of muscle lesion proved technically difficult to quantitate and was not measured in this study.

To further evaluate this methodology, we assessed retest reproducibility (i.e., the intra-observer coefficient of variability [CV]), inter-observer variability, and calculated intra-class correlation coefficients (ICCs) [20] in a subset of lesions (n = 20; half were PVL^+^ and half were PVL^−^, and lesions spanned a wide range) using two blinded observers. We also performed lesion measurements using an independent measurement method that utilized computer-assisted histomorphometric assessment of lesion area (ImageJ; open-source available from the NIH at http://rsb.info.nih.gov/ij/) [21]. Two blinded investigators with experience in mouse anatomy and lesion measurement independently assessed twenty lesions spanning a wide range of lesion sizes using the manual method (i.e., measurement of lesion length and width) and the computer-assisted histomorphometric method (i.e., ImageJ). Each observer then measured each lesion a second time after the lesions were shuffled to control for order effects. Lesion sizes were expressed as both area measurements (in mm^2^) and as a dimensionless ratio of left (i.e., PVL^+^) to right (i.e., PVL^−^). Intra-observer CVs (i.e., retest reproducibility) were between 6% and 11%, inter-observer CVs were 6% to 12%. ICCs were 0.882 for the manual method and 0.960 for the histomorphometric technique. The overall ICC between the two methods was 0.930.

### Immunofluorescent assays (IFA) and hematoxylin-eosin (H&E) stain

For IFA, the infected tissue was excised and fixed in 10% formalin (Medical Chemical Corporation) overnight. Paraffin embedding and H&E staining were performed by the Department of Pathology at Cedars-Sinai Medical Center. For IFA, tissue sections were deparaffinized and blocked with 5% goat serum in PBS with 0.05% Tween 20 (ISC Biosciences) (blocking buffer) for 1 hour at 37°C. After incubating samples with rabbit anti-LukS-PV antibody (diluted at 1∶200 in blocking buffer) at 37°C for 1 hour, slides were washed 5 min with PBS 3 times and incubated with corresponding FITC-conjugated secondary antibody (Sigma). One unit of Texas Red-phalloidin (Invitrogen) was used for counter staining per slide. After a final wash, tissue sections were mounted with Prolong AntiFade containing DAPI (Invitrogen). A minimum of six mice were used for each condition. Stained slides were examined using Olympus BX51 fluoresces microscope.

### Enzyme linked immunosorbent assay (ELISA)

Mouse MIP-2, RANTES, TNF-α, IL-1β, and KC (R & D Systems) specific ELISAs were performed according to the manufacturer's instructions. For PVL ELISA, plates were coated overnight at 4°C with known concentrations of rLukF-PV or test samples .The wells were blocked using 5% skim milk and 0.5% normal goat serum (Sigma) for an hour at 37°C. Rabbit anti-LukF-PVL anti-serum (1∶20,000 in the blocking buffer) was added to each well. After 1 hour at 37°C, the wells were washed 3 times with PBS plus 0.1% Tween-20 (wash buffer). A goat anti-rabbit IgG conjugated to HRP (Cell Signaling) (1∶5,000 in blocking buffer) was next added for an hour at 37°C, and after 5 washes, HRP activity was detected using TMB substrate (Fisher Scientific). Threshold detection level of the assay is 2 ng/ml. PVL ELISA has been validated using supernatant from mouse tissue infected with PVL^−^ *S. aureus*, spiked with known concentrations of rLukF-PV toxin.

### Myeloperoxidase (MPO) Assay

Animals were euthanized at 3 and 12 h post-infection, and the skin and muscle lesions were homogenized in 1 mL of PBS-Triton X-100 (0.5%) with protease inhibitor cocktails (Roche). The homogenized suspension was centrifuged at 15,000×*g* for 10 min and supernatants were collected and assayed for MPO activity according to the manufacturer's instructions using isolated murine polymorphonuclear cells as standard (Invitrogen).

### Immunoblot analysis

Overnight bacterial cultures were standardized by %T_540_ to a concentration of 10^9^ CFU/mL. The supernatants were collected by centrifugation and separated using Nu-PAGE system (Invitrogen). Proteins were blotted onto nitrocellulose membranes and probed with specific antibodies against LukS-PV, LukF-PV, and α-toxin (Sigma) and corresponding secondary antibodies conjugated to HRP (Cell Signaling Technology Inc.). Specific proteins were visualized using ECLplus (Amersham Biosciences).

### Statistical analysis

Data were analyzed using Prism 4.03 (Graphpad Software, Inc.). The two-tailed Wilcoxon test was used to compare paired samples. Unpaired samples were analyzed using Mann-Whitney test. Unless otherwise indicated, a *p* value less than 0.05 was considered significant, and noted in the figures. The Kruskal-Wallis test was used when three or more groups of data were compared. In bar graphs, n≥6 and results are presented as mean±SEM.

## Supporting Information

Figure S1 Mouse infection using S. aureus inocula of 107 and 108 CFU. CD1 mice were inoculated subcutaneous on one flank with ∼107 or 108 PVL+S. aureus, and on the opposite flank with the same inoculum of isogenic PVL- S. aureus. The injected strains were: CST5 PVL+/−, CST6 PVL+/−, and Newman+empty vector/Newman+PVL expression vector. Mice were sacrificed on day 3 post-infection. (A) and (B) Muscle lesion size and total CFU from mice injected with 107 CST5 PVL+/− or CST6 PVL+/−. (C) and (D) Muscle lesion size and total CFU from mice injected with 108 Newman+empty vector/Newman+PVL expression vector. * p<0.05, ** p<0.01.
(0.08 MB TIF)

Figure S2 Complementation studies. CD1 mice were inoculated subcutaneous on both flanks with ∼109 CFU WT CST5+empty vector, CST5 KO+empty vector, or CST5 KO+PVL expression vector. Mice were sacrificed on day 3 post-infection. (A) Muscle lesion size. (B) Skin lesion size. (C) Total tissue CFU. Please refer to Table S1 for detailed description of vectors. Graphs on the left show ratios of lesion sizes or ratios of CFU (PVL+∶PVL−) based on measurements from each individual mouse; graphs on the right show lesion sizes or CFU grouped according to bacterial strains. Note that there are only 6 data points showing WT/KO+PVL ratios: Only 6 mice were injected with paired WT CST5+empty vector on one flank and CST5 KO+PVL expression vector on the opposite flank. ** p<0.01.
(0.11 MB TIF)

Figure S3 H&E stain of infected tissues. CD1 mice were infected with either PVL+ or isogenic PVL- S. aureus as previously described. Shown are H&E stainings of uninfected and infected tissues (at day 3 post-infection). E+D:epidermis-dermis layer, SA: S. aureus, and M:muscle.
(5.04 MB TIF)

Figure S4 Effect of innate immunity and host background on PVL virulence function. Ten to twelve week old CD1, C57BL/6, BALB/c, and SKH1 mice were infected on opposite flanks with either PVL+CST5 or isogenic PVL- CST5. (A) Muscle lesion size and CFU on day 3 post-infection. (B) Tissue MPO level at 3 and 12 h after subcutaneous infection of CD1 mice with CST5+/−PVL. Controls consisted of PBS injected mice (negative control) and LPS injected mice (positive control). * p<0.05.
(0.07 MB TIF)

Table S1 Strains and plasmids used in this study.
(0.06 MB DOC)
