# Supplementary figures and images for: Staphylococcus aureus Panton-Valentine Leukocidin Contributes to Inflammation and Muscle Tissue Injury
Source: PLoS One. 2009 Jul 27;4(7):e6387. doi: 10.1371/journal.pone.0006387 (PMC2711303; doi:10.1371/journal.pone.0006387)

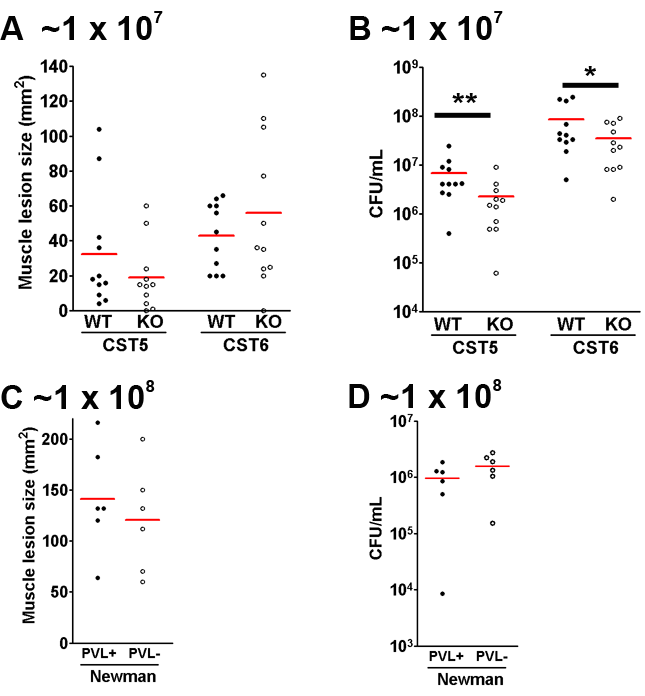

Supplement: Figure S1 — Mouse infection using S. aureus inocula of 107 and 108 CFU. CD1 mice were inoculated subcutaneous on one flank with ∼107 or 108 PVL+S. aureus, and on the opposite flank with the same inoculum of isogenic PVL- S. aureus. The injected strains were: CST5 PVL+/−, CST6 PVL+/−, and Newman+empty vector/Newman+PVL expression vector. Mice were sacrificed on day 3 post-infection. (A) and (B) Muscle lesion size and total CFU from mice injected with 107 CST5 PVL+/− or CST6 PVL+/−. (C) and (D) Muscle lesion size and total CFU from mice injected with 108 Newman+empty vector/Newman+PVL expression vector. * p<0.05, ** p<0.01. (0.08 MB TIF) [file pone.0006387.s001.tif]

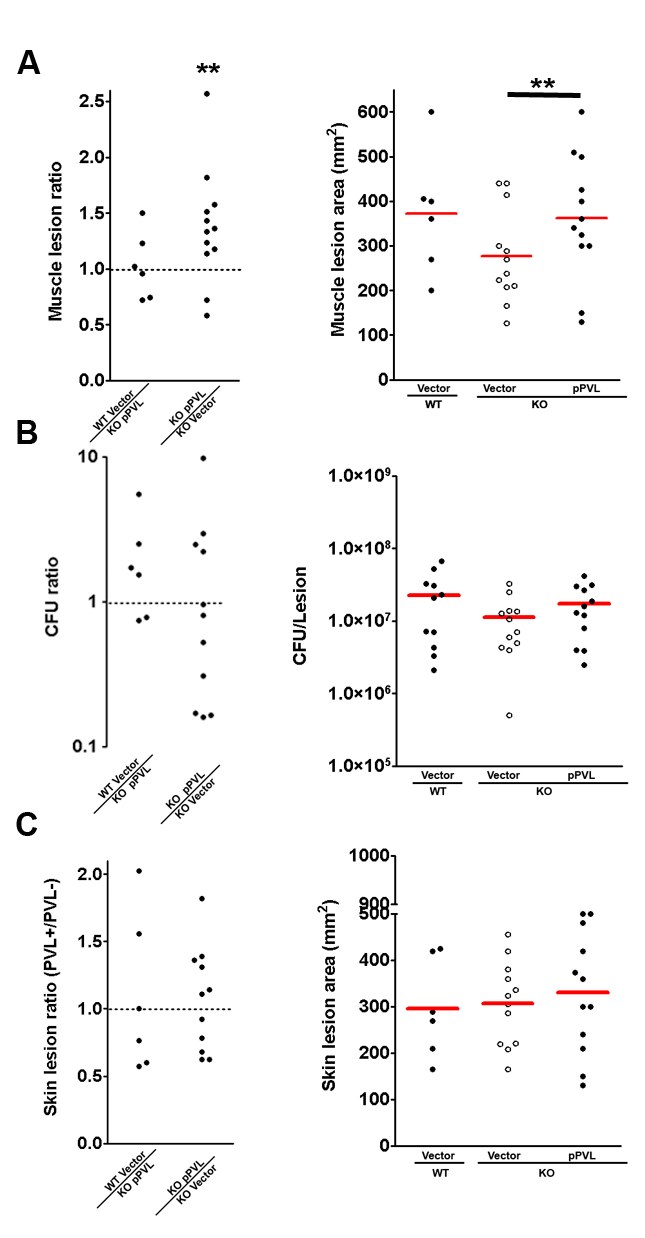

Supplement: Figure S2 — Complementation studies. CD1 mice were inoculated subcutaneous on both flanks with ∼109 CFU WT CST5+empty vector, CST5 KO+empty vector, or CST5 KO+PVL expression vector. Mice were sacrificed on day 3 post-infection. (A) Muscle lesion size. (B) Skin lesion size. (C) Total tissue CFU. Please refer to Table S1 for detailed description of vectors. Graphs on the left show ratios of lesion sizes or ratios of CFU (PVL+∶PVL−) based on measurements from each individual mouse; graphs on the right show lesion sizes or CFU grouped according to bacterial strains. Note that there are only 6 data points showing WT/KO+PVL ratios: Only 6 mice were injected with paired WT CST5+empty vector on one flank and CST5 KO+PVL expression vector on the opposite flank. ** p<0.01. (0.11 MB TIF) [file pone.0006387.s002.tif]

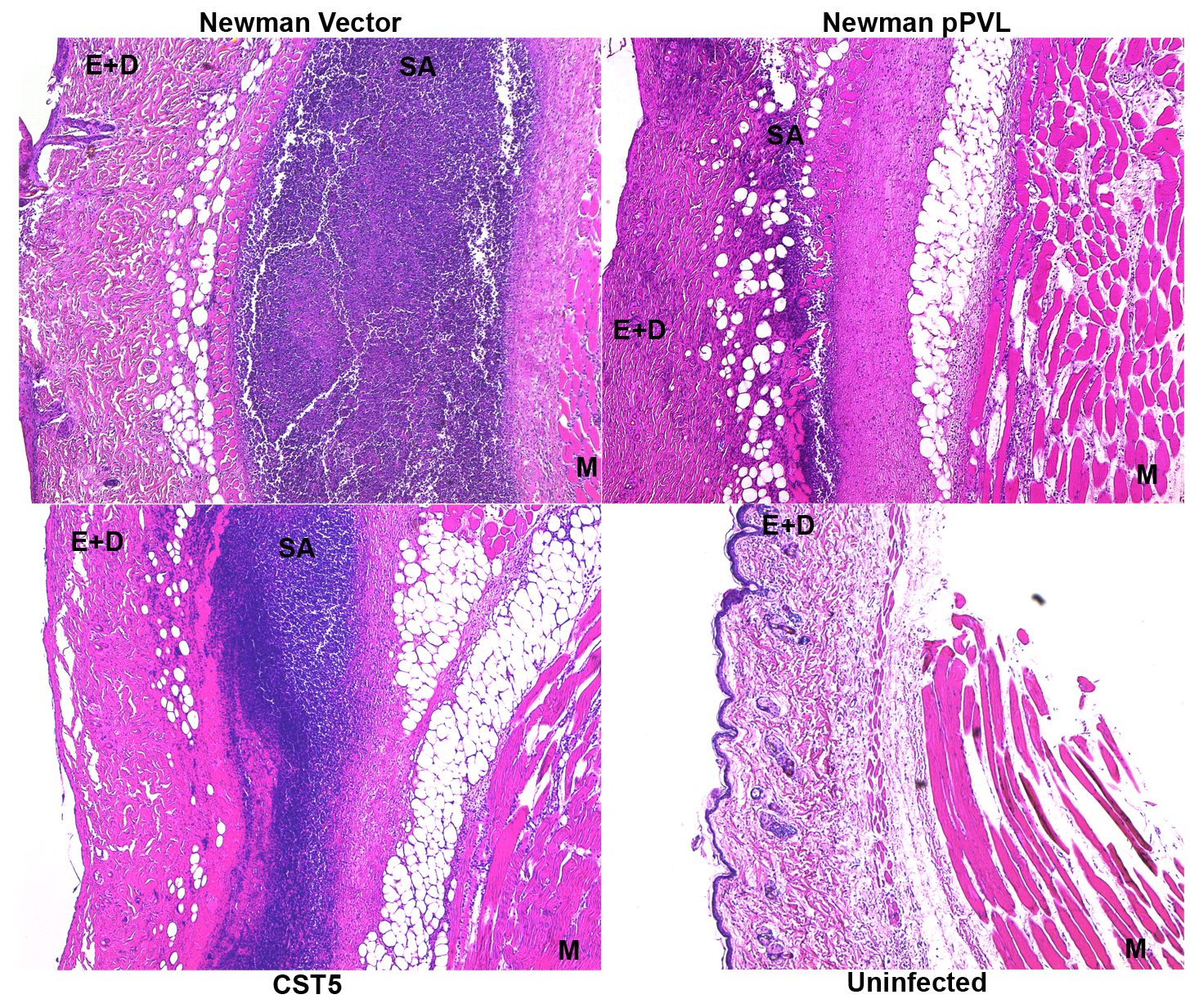

Supplement: Figure S3 — H&E stain of infected tissues. CD1 mice were infected with either PVL+ or isogenic PVL- S. aureus as previously described. Shown are H&E stainings of uninfected and infected tissues (at day 3 post-infection). E+D:epidermis-dermis layer, SA: S. aureus, and M:muscle. (5.04 MB TIF) [file pone.0006387.s003.tif]

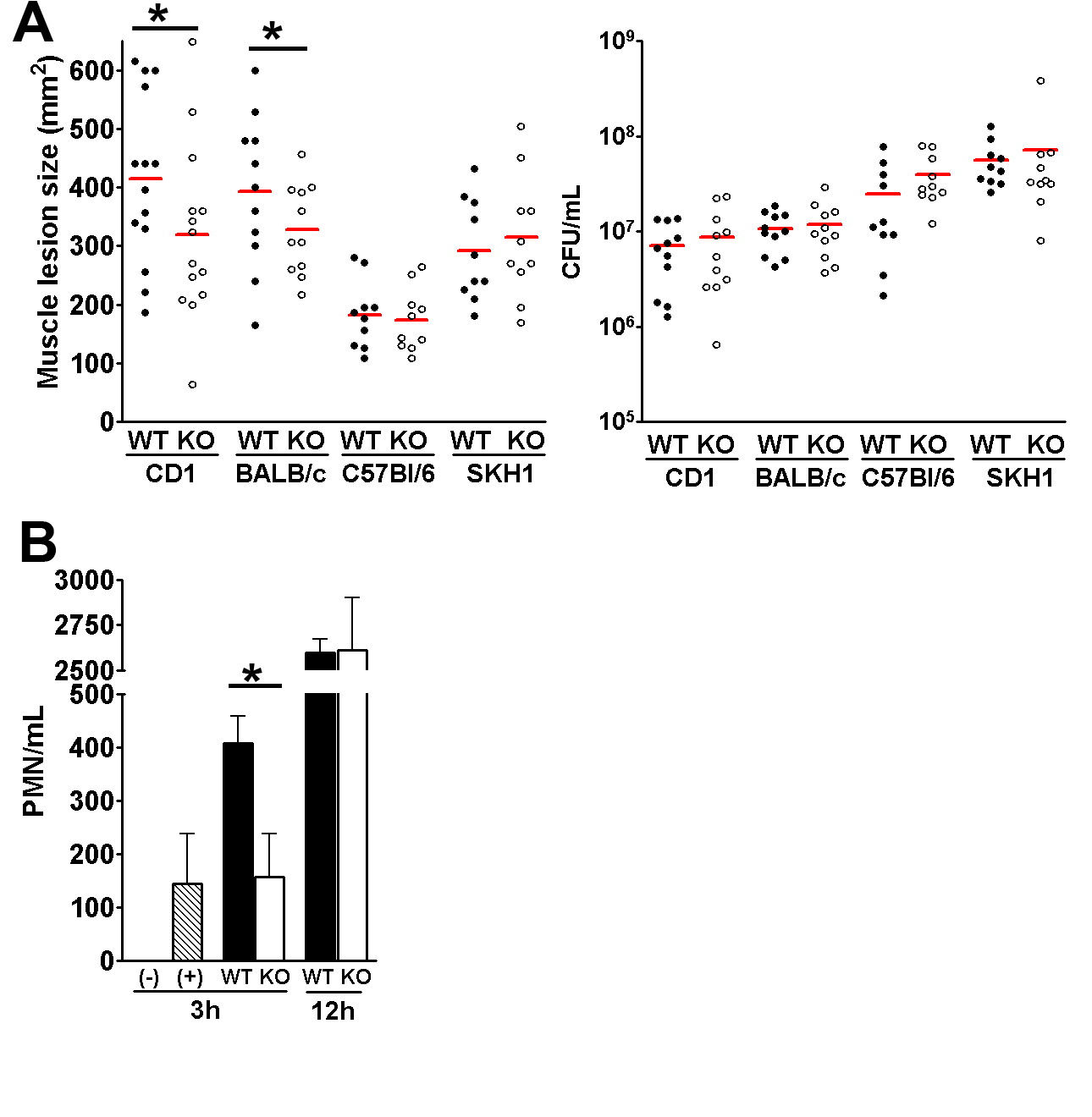

Supplement: Figure S4 — Effect of innate immunity and host background on PVL virulence function. Ten to twelve week old CD1, C57BL/6, BALB/c, and SKH1 mice were infected on opposite flanks with either PVL+CST5 or isogenic PVL- CST5. (A) Muscle lesion size and CFU on day 3 post-infection. (B) Tissue MPO level at 3 and 12 h after subcutaneous infection of CD1 mice with CST5+/−PVL. Controls consisted of PBS injected mice (negative control) and LPS injected mice (positive control). * p<0.05. (0.07 MB TIF) [file pone.0006387.s004.tif]
