# Supplementary material for: Staphylococcus aureus Panton-Valentine Leukocidin Contributes to Inflammation and Muscle Tissue Injury
Source: PLoS One. 2009 Jul 27;4(7):e6387. doi: 10.1371/journal.pone.0006387 (PMC2711303; doi:10.1371/journal.pone.0006387)
Supplement: Table S1 — Strains and plasmids used in this study. (0.06 MB DOC) [file pone.0006387.s005.doc]

**Table S1. Strains and plasmids used in this study**

| Name | Description | Reference/Source |
| --- | --- | --- |
| Bacteria strains |  |  |
| *Staphylococcus aureus* |  |  |
| CST5 | Clinical isolate. SCCmec IV, ST8, USA300 | (1) |
| CST6 | Clinical isolate. SCCmec IV, ST8, USA300 | (1) |
| RN4220 | Accepts foreign DNA (r^–^) | (2) |
| Newman | Laboratory strain | **Goetz, F.** |
| CST151 | CST5, *luk-*PV 2 stop codons-*erm*^R^ | This study |
| CST153 | CST6, *luk-*PV 2 stop codons-*erm*^R^ | This study |
| BD0299 | SF8300, *luk*-PV::*spec*^R^ | This study |
| CST176 | CST5, pDT144 | This study |
| CST178 | CST151, pDT144 | This study |
| CST181 | CST151, pDT145 | This study |
| Newman | pDCErm | This study |
| Newman | pDCErm+PVL | This study |
| Plasmids |  |  |
| pDT144 | 3.9 kb pCR2.1 BamHI fragment in pDL278 | This study |
| pDT145 | pDT144 + 2.2 kb PVL DNA fragment | This study |
| pDCErm | Shuttle vector | (3) |
| pDCErm + PVL | 2.2 kb PVL expression fragment in pDCErm | This study |
| pCR2.1 | PCR cloning vector | Invitrogen |
| pET151 | Expression vector | Invitrogen |
| pDL278 | Shuttle vector | (4) |
| pET151LukS | rLukS expression vector | This study |
| pET151LukF | rLukF expression vector | This study |

MLST, multilocus sequence type; SSC, staphylococcal cassette chromosome; ST, sequence type

**REFERENCES**

1. Miller, L.G., Perdreau-Remington, F., Rieg, G., Mehdi, S., Perlroth, J., Bayer, A.S., Tang, A.W., Phung, T.O., and Spellberg, B. 2005. Necrotizing fasciitis caused by community-associated methicillin-resistant Staphylococcus aureus in Los Angeles. *N Engl J Med* 352:1445-1453.

2. Kreiswirth, B.N., Lofdahl, S., Betley, M.J., O'Reilly, M., Schlievert, P.M., Bergdoll, M.S., and Novick, R.P. 1983. The toxic shock syndrome exotoxin structural gene is not detectably transmitted by a prophage. *Nature* 305:709-712.

3. Jeng, A., Sakota, V., Li, Z., Datta, V., Beall, B., and Nizet, V. 2003. Molecular genetic analysis of a group A Streptococcus operon encoding serum opacity factor and a novel fibronectin-binding protein, SfbX. *J Bacteriol* 185:1208-1217.

4. LeBlanc, D.J., and Lee, L.N. 1991. Replication functions of pVA380-1. In *Genetics and molecular biology of streptococci, enterococci, and lactococc*. G.M. Dunny, P.P. Cleary, and L.L. McKay, editors. Washington, D.C.: American Society for Microbiology. 224-227.
